# Protective role of complement signaling in Kawasaki disease vasculitis

**DOI:** 10.1172/jci.insight.203997

**Published:** 2026-05-12

**Authors:** Asli E. Atici, Begüm Kocatürk, Benjamin L. Ross, Emily A. Aubuchon, Rebecca A. Porritt, Thacyana T. Carvalho, Takahiro Namba, Youngho Lee, Magali Noval Rivas, Moshe Arditi

**Affiliations:** 1 Department of Pediatrics, Cedars-Sinai Guerin Children’s,; 2 Infectious and Immunologic Diseases Research Center (IIDRC) and Department of Biomedical Sciences, and; 3 Smidt Heart Institute, Cedars-Sinai Health Sciences University, Los Angeles, California, USA.

**Keywords:** Immunology, Inflammation, Vascular biology, Complement, Macrophages

## Abstract

Kawasaki disease (KD) is an acute febrile systemic vasculitis of unknown etiology and the leading cause of acquired heart disease among children. Complement activation has long been observed in patients with acute KD; however, its contribution to disease development remains unknown. Here, using publicly available datasets, we showed that patients with acute KD exhibited higher expression of complement products in whole blood, consistent with the activation of the complement pathway. Similarly, in the *Lactobacillus casei* cell wall extract (LCWE) murine model of KD, LCWE injection induced increased expression of complement products in cardiovascular tissues, suggestive of activation of the complement pathways. C3-deficient mice or WT mice treated with the complement C5a receptor 1 (C5ar1) antagonist developed significantly more severe LCWE-induced cardiovascular lesions and vasculitis. Furthermore, we observed that LCWE binds to serum C3, an opsonizing factor that labels microbial targets for clearance, and LCWE deposition in the liver was significantly higher in C3-deficient mice compared with WT mice. Overall, our data indicate that blocking the complement system significantly exacerbates LCWE-induced KD vasculitis, likely by impairing C3-mediated clearance of LCWE. These data suggest that the complement pathway may play a protective role in KD pathogenesis by promoting clearance of a potential bacterial or viral trigger of KD.

## Abstract 2

This study identifies the complement C3–C5 axis as a protective pathway in mouse KD that limits disease severity by facilitating clearance of microbial triggers.

## Graphical abstract

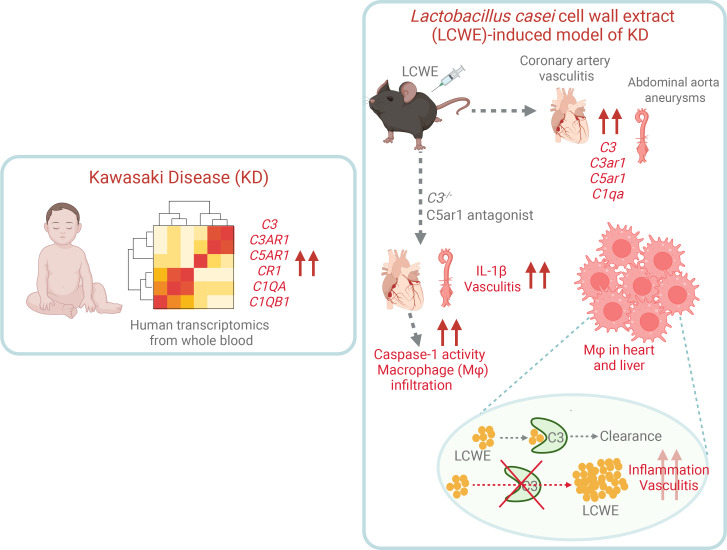

## Introduction

Kawasaki disease (KD) is an acute febrile illness and systemic vasculitis of unknown etiology, affecting young children and causing coronary abnormalities and aneurysms in up to 25% of untreated patients (1, 2). Despite years of research, the etiological trigger of KD remains unidentified, although it is suspected to be an infectious agent (1, 2). Intravenous immunoglobulin (IVIG) treatment and high-to-moderate dose aspirin within the first 10 days of disease onset reduce inflammation and decrease the incidence of coronary aneurysms to 3%–5% (2). However, up to 20% of patients with KD are IVIG-resistant and at greater risk for developing coronary abnormalities and aneurysms (3, 4). After the acute disease period, remodeling of the affected vessels and myocardium can lead to long-term cardiovascular sequelae, such as coronary artery stenosis, myocardial ischemia, and fibrosis (5, 6). Although human and murine research has highlighted IL-1 as central to the formation of KD cardiovascular lesions, clarifying KD pathophysiology is needed to uncover new treatment targets (7–12).

The complement system is a set of plasma and intracellular proteins that assemble into membrane attack complexes (MACs) to clear microorganisms or infected and damaged cells (13). The 3 arms of the complement pathway (classical, alternative, and mannose-binding lectin [MBL]) converge on the activation of C3 and C5, inducing complement protein deposition on the pathogen wall for clearance (14). Importantly, complement components can exacerbate proinflammatory signaling and the production of proinflammatory mediators, such as IL-1β (15, 16). Complement fragments operate through specific C3a and C5a_1_ receptors (C3ar1 and C5ar1) and induce inflammation through the local recruitment of phagocytic cells (17, 18). Notably, the central complement component, C3, is an opsonizing factor that covalently labels its targets for clearance by phagocytes (19). Early studies of KD detected individual complement breakdown products (C3d, C4d, Bb, MAC) in plasma from patients with KD and concluded that the disease process involves complement activation, mainly classical/lectin involvement, rather than simple consumption of intact C3/C4 (20). Several studies have shown increased expression of C3 and its receptor in patients with acute KD (21–23). More recent plasma studies report changes in factor B (alternative pathway), C5a, and other activation fragments during the acute phase of KD, suggesting that more than one pathway can be engaged in the course of the disease (24). Furthermore, MBL is a crucial arm of the complement system, which binds to mannose on the surface of pathogens to induce opsonization (25, 26). Several genetic and association studies report a link between *MBL/MBL2* polymorphisms and KD susceptibility and with coronary complications in some patient cohorts, indicating a possible role for lectin-pathway biology in KD pathogenesis (27–29). Furthermore, whole-blood transcriptomic data showed significantly increased expression of complement C3b/C4 receptor 1 (*CR1*) in patients with acute KD compared with healthy controls (HCs) (21). Despite all of these studies showing complement activation in patients with acute KD, the functional contribution of the complement pathway(s) to disease development remains unknown.

Although no animal model can fully mimic human disease, the *Lactobacillus casei* cell wall extract (LCWE) murine model of KD vasculitis closely mirrors the important histological and immunological aspects of human KD and is a widely accepted model in the KD research community (10, 30, 31). This model recapitulates the 3 linked pathological processes observed in human KD, including luminal myofibroblast proliferation in the coronary arteries (30, 32). In addition to causing inflammation, particularly in the coronary arteries, human KD is also associated with dilation and aneurysms, including abdominal aortic aneurysm (33–36). In support of this finding, a single i.p. injection of LCWE in mice induces aneurysm formation in the infrarenal region of the abdominal aorta, similar to that observed in patients with KD (11). NLR family pyrin domain-containing 3 (NLRP3) inflammasome activation, IL-1β production, and the rapid influx of innate immune cells, neutrophils, and macrophages into cardiovascular tissues are hallmarks of LCWE-induced KD vasculitis (8, 9, 11). Targeting the IL-1 pathway, either genetically or pharmacologically, reduces the severity of LCWE-induced vasculitis, indicating that LCWE-induced KD depends on the NLRP3/IL-1 axis (9–11).

In this study, we observed increased levels of complement system fragments and receptors in the whole blood of patients with acute KD and in cardiovascular tissues of LCWE-injected mice. Furthermore, knocking out C3 or blocking C5ar1 with an antagonist significantly exacerbated the development of cardiovascular lesions in this KD vasculitis model. We also demonstrated C3 binding to LCWE and that C3 deficiency increased the accumulation of fluorescently labeled LCWE in tissue-resident macrophages. Taken together, our findings suggest that an intact complement system plays a protective and regulatory role during the early immune response in this experimental KD vasculitis model.

## Results

### Increased expression of complement system proteins and receptors in whole blood of patients with acute KD and cardiovascular lesions of LCWE-injected mice.

**Figure 1:**
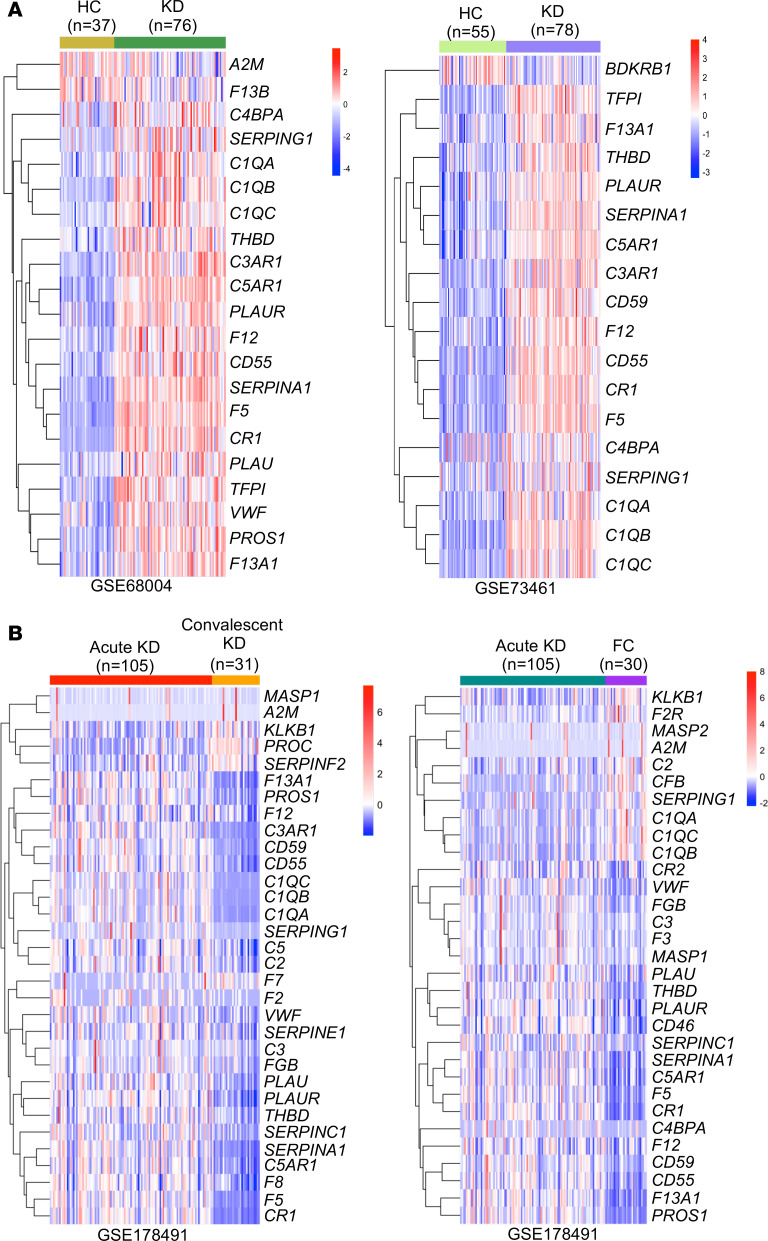
Increased expression of complement pathway fragments and receptors in patients with acute KD. (**A**) Heatmaps illustrating the expression of genes involved in complement pathway activation in whole blood from patients with acute KD compared with healthy controls (HCs) (GSE68004 and GSE73461). Color gradient represents relative expression to the mean of each row. (**B**) Heatmaps illustrating the expression of genes involved in complement pathway activation in whole blood from patients with acute KD compared with convalescent KD or febrile controls (FCs) (GSE178491). Color gradient represents relative expression to the mean of each row. Differential expression was analyzed with GEO2R, and only genes of interest with an FDR < 0.05 are represented.

**Figure 2:**
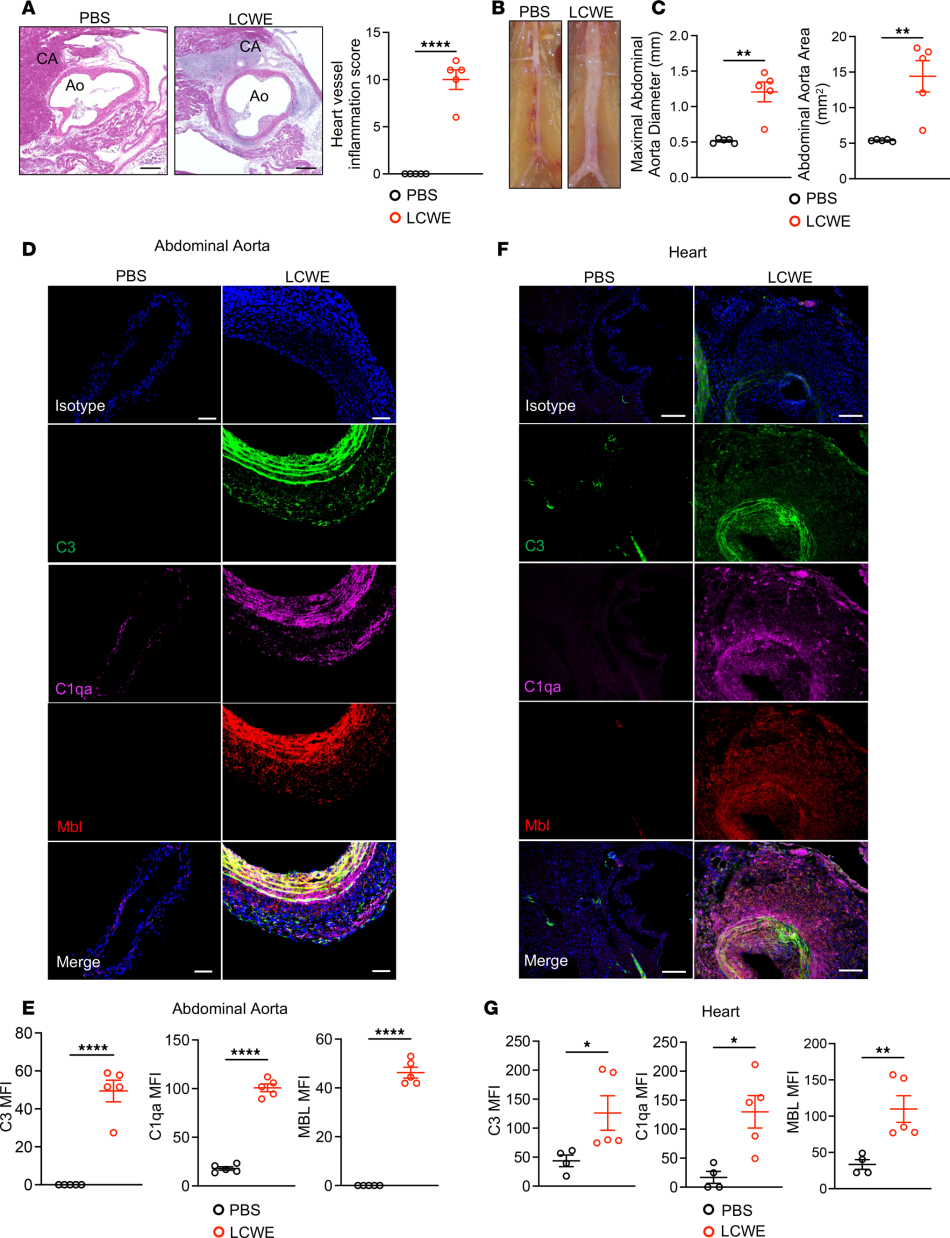
Increased protein levels of complement arms in LCWE-induced murine KD. (**A**) Representative H&E-stained heart sections and heart vessel inflammation scores of WT mice injected with either PBS or LCWE at 2 weeks after injection (*n* = 5/group). Scale bars: 500 μm. (**B** and **C**) Representative pictures of the abdominal aorta areas (**B**) and maximal abdominal aorta diameter and abdominal aorta area measurements (**C**) of WT mice injected with PBS or LCWE at 2 weeks after injection. (**D**) Immunofluorescent staining of C3 (green), C1qa (purple), and Mbl (red) from abdominal aortas of PBS- or LCWE-injected mice at 2 weeks (*n* = 5/group). DAPI (blue) was used to stain nuclei. Scale bars: 50 μm. (**E**) MFI of C3, C1qa, and Mbl staining from **D**. (**F**) Immunofluorescent staining of C3 (green), C1qa (purple), and Mbl (red) from hearts of PBS- or LCWE-injected mice at 2 weeks (*n* = 4, 5/group). DAPI (blue) was used to stain nuclei. Scale bars: 50 μm. (**G**) MFI of C3, C1qa, and Mbl staining from **F**. Data are presented as mean ± SEM. *P < 0.05, **P < 0.01, and ****P < 0.001 by unpaired 2-tailed *t* test (**A**–**C**, **E**, and **G**). Ao, aorta; CA, coronary artery.

We first examined the expression of the overall complement system genes in publicly available datasets generated from whole blood of patients with acute KD and HCs (NCBI’s Gene Expression Omnibus [GEO] GSE73461 and GSE68004) (37, 38). The Kyoto Encyclopedia of Genes and Genomes (KEGG) Complement and Coagulation Cascades gene set (hsa04610) was used as a reference (Supplemental Figure 1; supplemental material available online with this article; https://doi.org/10.1172/jci.insight.203997DS1). Of the 69 genes identified, 21 in GSE68004 and 18 in GSE73461 were differentially expressed. The expression of genes involved in the 3 arms of the complement pathway, including *C3AR1* and *C5AR1* and classical pathway components *C1QA, C1QB,* and *C1QC,* was increased in patients with acute KD compared with HCs in both datasets (Figure 1A). We next used another dataset generated from whole blood of acute KD, convalescent KD, and febrile controls (GSE178491) (39). We identified 32 differentially expressed genes (DEGs) in patients with acute KD compared with convalescent patients with KD and 31 DEGs in patients with acute KD compared with febrile controls (Figure 1B). Similar to the other datasets, expression of *C5AR1*, *C3*, and *CR1* was upregulated in patients with acute KD compared with both convalescent KD and febrile controls. Notably, expression of *C1QA*, *C1QB,* and *C1QC* was upregulated in acute KD compared with the convalescent phase (Figure 1B). Next, we analyzed a single-cell RNA-Seq (scRNA-Seq) dataset, profiling PBMCs from patients with acute KD and HCs (GSE168732) (40). Expression of *C1QA*, *C3AR1*, *C5AR1,* and *CR1* was increased in mainly monocytes and neutrophils during acute KD compared with HCs, suggesting activation of the complement system in these cells (Supplemental Figure 2A). A single injection of LCWE induces cardiovascular inflammation and abdominal aorta aneurysms in WT mice (8–11, 41, 42) (Figure 2, A–C). First, we examined the protein levels of C3, C1qa, and Mbl in the cardiovascular tissues of mice injected in this model. Immunofluorescence staining showed significantly higher levels of all 3 proteins in the abdominal aortas and hearts of LCWE-injected KD mice compared with controls (Figure 2, D–G).

**Figure 3:**
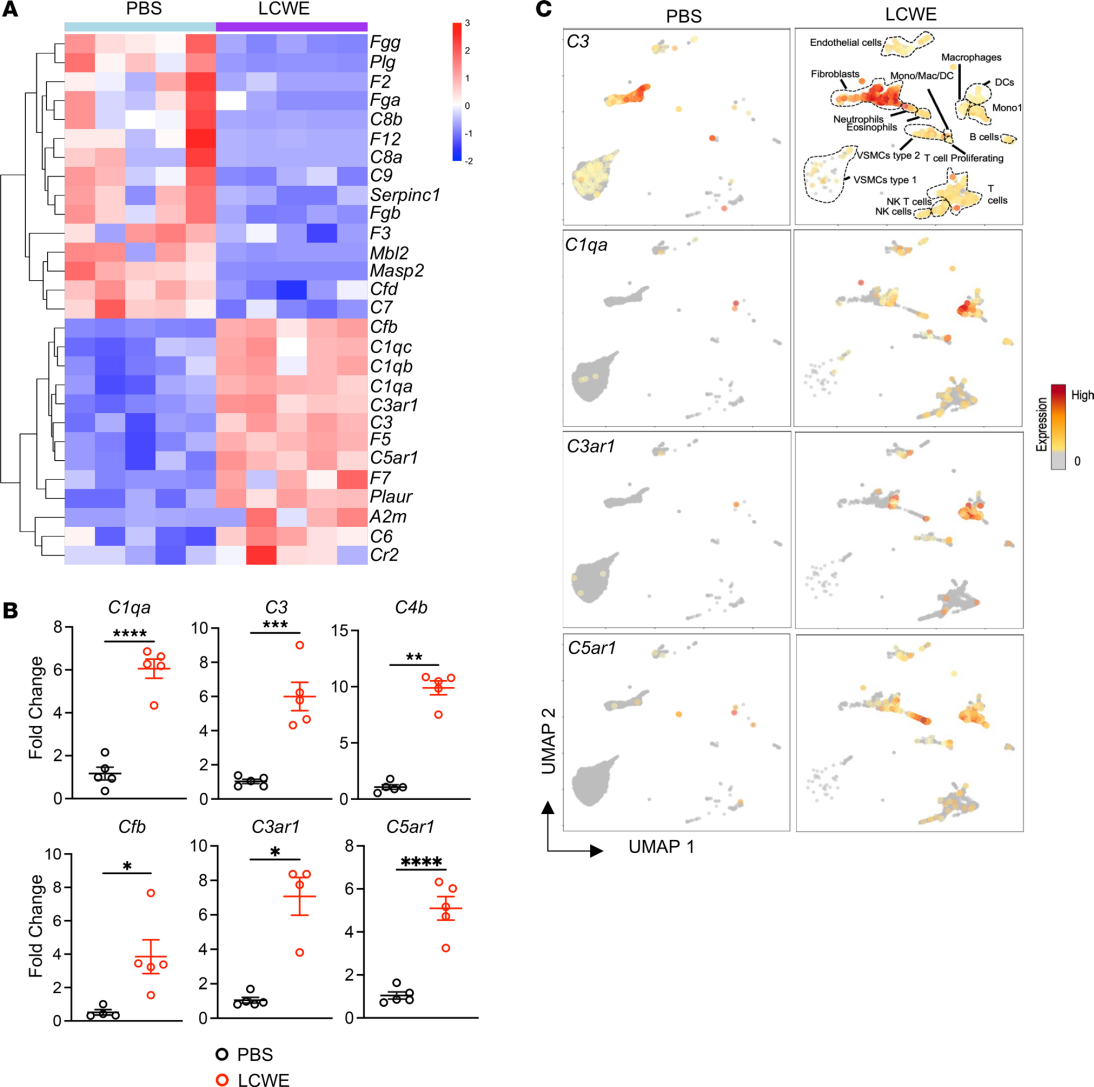
Complement pathway expression in abdominal aortas from LCWE-injected mice. (**A**) Differentially regulated genes (adjusted *P* < 0.05, fold change > 2) related to the complement pathway analyzed from abdominal aortas of PBS- or LCWE-injected mice at 2 weeks after injection (GSE141072). (**B**) mRNA expression of complement signature genes *C3*, *C1qa*, *C3ar1,* and *C5ar1* measured by qRT-PCR from abdominal aortas of PBS- and LCWE-injected mice, normalized to *Hprt* (*n* = 5/group). (**C**) UMAP plot of all abdominal aorta cells isolated from PBS-injected (*n* = 5,386 cells, blue) and LCWE-injected (*n* = 5,091cells, red) WT mice. Each panel shows a gradient expression of selected genes related to the complement pathway. Grey-yellow-red gradient: min-max normalization of CP10K expression (GSE178765). Data are presented as mean ± SEM. *P < 0.05, **P < 0.01, ***P < 0.001, and ****P < 0.001 by unpaired 2-tailed *t* test (**A** and **B**). CP10K indicates counts per 10,000 reads. VSMC, vascular smooth muscle cell; Mono: monocytes.

**Figure 4:**
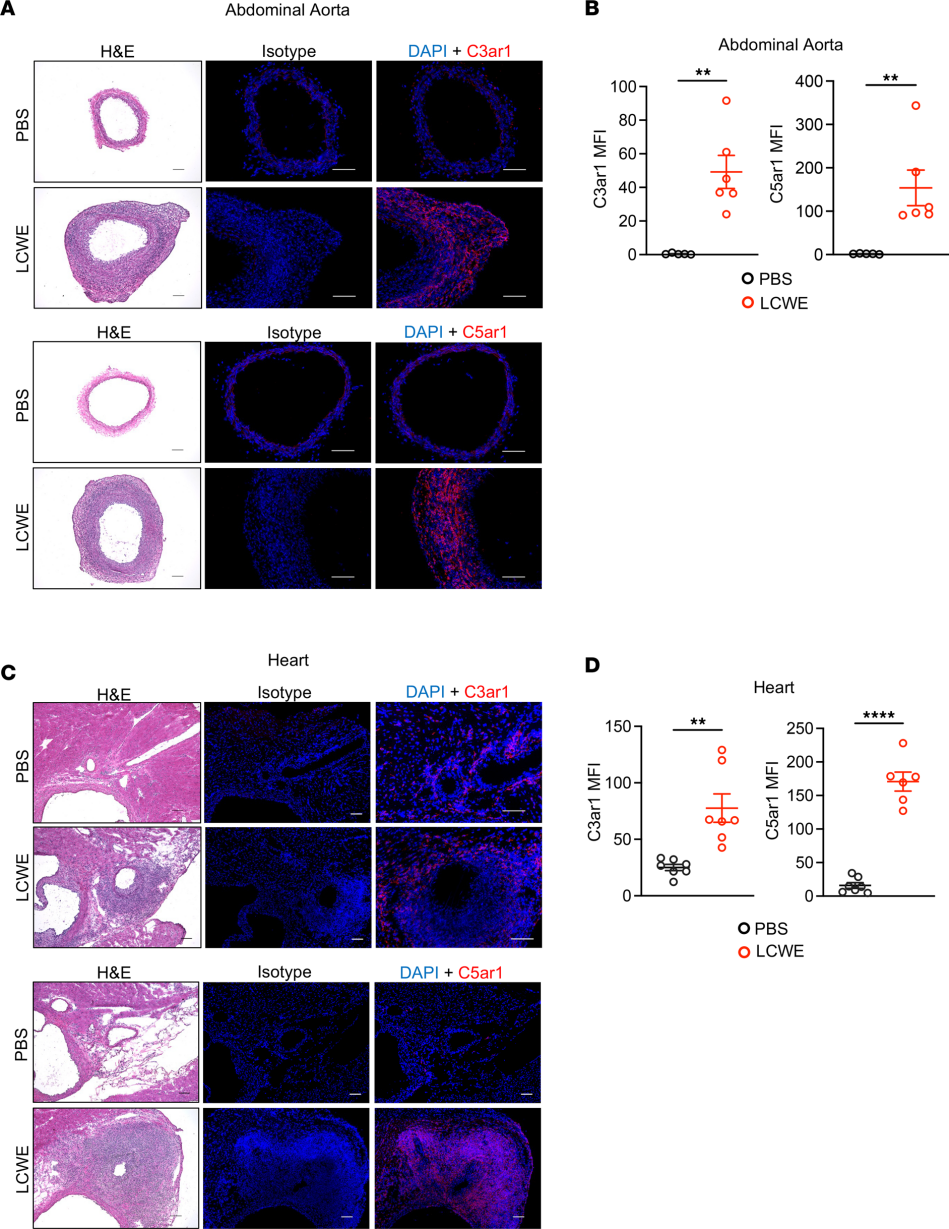
Protein levels of complement pathway receptors are elevated in cardiovascular tissues of LCWE-induced KD mice. (**A**) Representative H&E-stained sections or immunofluorescent staining of C3ar1 (red) and C5ar1 (red) from abdominal aortas of PBS- or LCWE-injected mice at 2 weeks. DAPI (blue) was used to stain nuclei (*n* = 5, 6/group). Scale bars: 100 μm. (**B**) MFI of C3ar1 and C5ar1 staining from **A**. (**C**) Representative H&E-stained sections or immunofluorescent staining of C3ar1 (red) and C5ar1 (red) from hearts of PBS- or LCWE-injected mice at 2 weeks (*n* = 6, 7/group). DAPI (blue) was used to stain nuclei. Scale bars: 100 μm. (**D**) MFI of C3ar1 and C5ar1 staining from **C**. Data are presented as mean ± SEM. **P < 0.01 and ****P < 0.001 by unpaired 2-tailed *t* test (**B** and **D**).

We next analyzed the expression levels of genes involved in the complement pathway in abdominal aortic tissues of PBS or LCWE-injected WT mice using a published bulk RNA-Seq dataset (GSE141072) (9) (Figure 3A). Out of 59 murine orthologs of human complement genes (Supplemental Figure 1B), we identified 28 DEGs in this dataset (Figure 3A). Similar to the transcriptomic results in whole blood of patients with KD, we observed a significant increase in *C3ar1*, *C5ar1*, *C3*, *C1qa*, *C1qb,* and *C1qc* in abdominal aortas of LCWE-injected mice compared with PBS controls (Figure 3A). We also confirmed the increase in mRNA expression of selected complement pathway genes by quantitative real-time PCR (qRT-PCR) in the abdominal aortas of LCWE-injected mice compared with PBS control mice in a separate cohort (Figure 3B). In addition, we observed increased protein levels of cleaved C3 fragments (C3b/C3c) and Mbl in the abdominal aorta tissues of LCWE-injected mice by Western blot (Supplemental Figure 3A, B). To determine the cellular expression of complement system genes during LCWE-induced KD vasculitis, we next examined a published scRNA-Seq dataset generated from abdominal aorta tissues of PBS- and LCWE-injected mice (GSE178765) (Figure 3C) (41). We observed expression of *C3*, *C1qa*, *C3ar1,* and *C5ar1* in immune cells infiltrating the abdominal aorta after LCWE-injection, specifically in monocytes, macrophages, DCs, B cells, T cells, and proliferative and type 2 vascular smooth muscle cells (VSMCs) (Figure 3C). Furthermore, we used a spatial transcriptomics dataset (GSE178799) to characterize the spatial organization of complement-related genes in LCWE-induced heart tissue lesions (41). Around the inflamed coronary artery of LCWE-injected heart tissue, most of the infiltrating cells were monocytes, DCs, and macrophages (Supplemental Figure 4). These infiltrating immune cells spatially overlapped with high expression of *C1qa*, *C3ar1*, *C5ar1,* and *C3* (Supplemental Figure 4). Since complement fragments induce local inflammation and operate through specific C3 and C5 receptors, we next performed immunofluorescence staining for C3ar1 and C5ar1 in the cardiovascular tissues of PBS and LCWE-injected mice. The protein levels of both receptors were also significantly higher in LCWE-induced cardiovascular lesions (Figure 4, A–D). Together, these data indicate that the expression of complement fragments and their receptors is upregulated in patients with acute KD and in LCWE-injected mice that develop a KD-like vasculitis.

### C3 deficiency or blockade of the C5a receptor significantly exacerbates LCWE-induced KD vasculitis.

**Figure 5:**
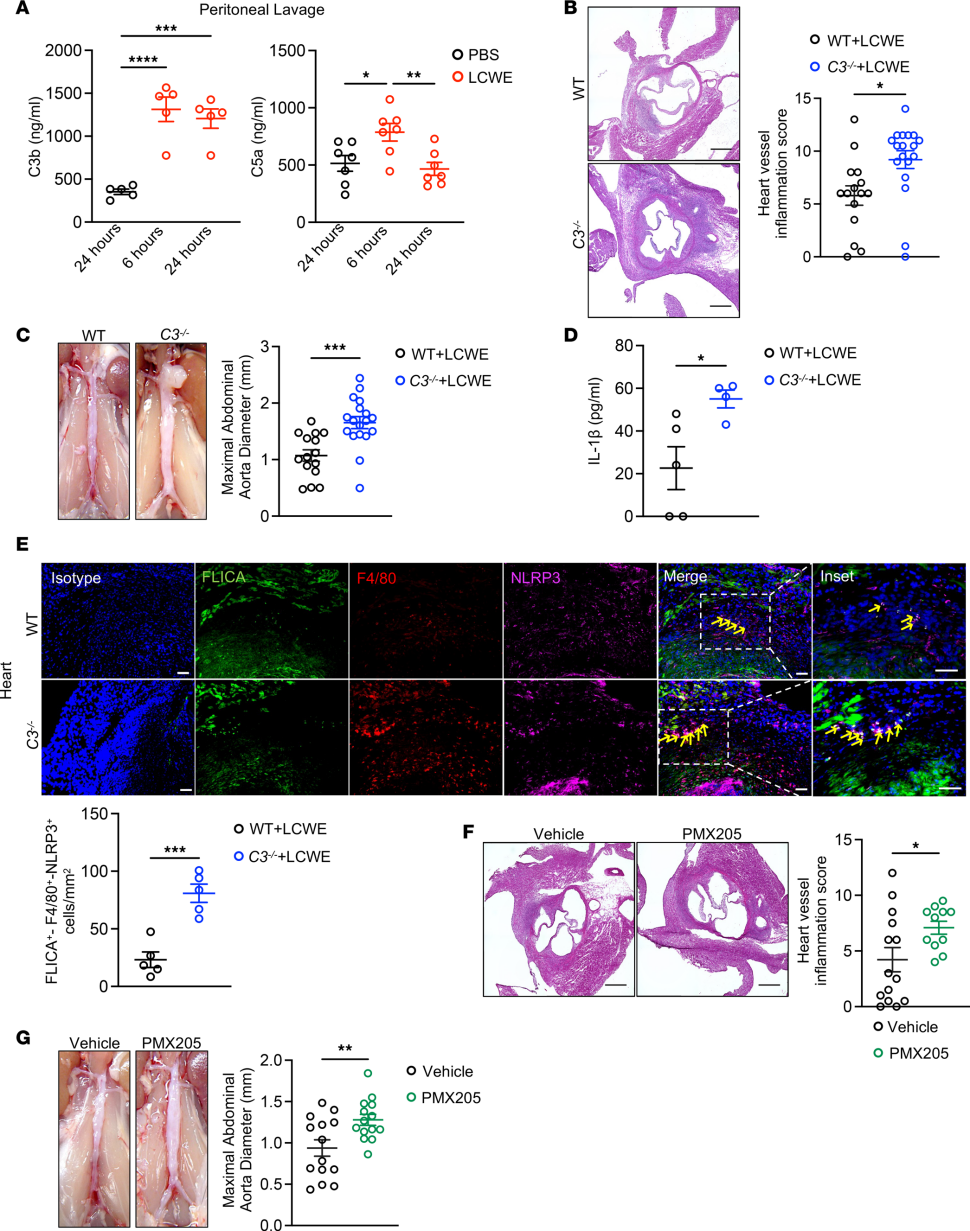
C3 deficiency or C5a receptor blockade exacerbates murine KD vasculitis. (**A**) C3b and C5a levels in the peritoneal lavage of WT mice injected with PBS or LCWE for 6 or 24 hours (*n* = 5–7/group). (**B**) Representative H&E-stained heart sections and heart vessel inflammation scores of LCWE-injected WT and *C3^–/–^* mice at 2 weeks after injection (*n* = 15–18/group). Scale bars: 500 μm. (**C**) Representative pictures of the abdominal aorta areas and maximal abdominal aorta diameter measurements of LCWE-injected WT and *C3^–/–^* mice at 2 weeks after injection (*n* = 15–18/group). (**D**) IL-1β measurements in the peritoneal lavage of WT and *C3^–/–^* mice injected with PBS or LCWE 24 hours after injection (*n* = 4, 5/group). (**E**) Representative images and quantification of FLICA (green), F4/80 (red), and NLRP3 (purple) staining in hearts from LCWE-injected WT and *C3^–/–^* mice at 2 weeks after injection (*n* = 5/group). DAPI (blue) was used to stain nuclei. Yellow arrows indicate triple positive cells. Scale bars: 50 μm. (**F**) Representative H&E-stained heart sections and heart vessel inflammation scores of LCWE-injected WT mice treated with vehicle or PMX205 (C5ar1 antagonist) at 2 weeks after injection (*n* = 11–14/group). Scale bars: 500 μm. (**G**) Representative pictures of the abdominal aorta areas and maximal abdominal aorta diameter measurements of LCWE-injected WT mice treated with vehicle or PMX205 at 2 weeks after injection (*n* = 11–14/group). Data are pooled from 2 separate experiments in **B**, **C**, **F**, and **G**. Data are presented as mean ± SEM. *P < 0.05, **P < 0.01, ***P < 0.001, and ****P < 0.001 by 1-way ANOVA with Tukey’s (**A**) and unpaired 2-tailed *t* test (**B**–**G**).

Since all 3 arms of the complement system converge on C3 and C5, we next sought to determine their contributions to cardiovascular lesion development in LCWE-induced KD vasculitis. We first measured C3b and C5a levels in the peritoneal lavage of WT mice injected with PBS or LCWE at 6 and 24 hours after injection. Both C3b and C5a were significantly increased in the peritoneal fluid at 6 hours after LCWE (Figure 5A). At 24 hours after LCWE injection, C3b levels were still high, whereas C5a levels had returned to baseline (Figure 5A). These data suggest activation of C3 and C5 early after LCWE injection in this vasculitis model.

To test the functional impact of C3 on KD vasculitis in vivo, we next injected WT and C3-deficient (*C3^–/–^*) mice with LCWE and collected tissues at 2 weeks after injection. Strikingly, C3 deficiency led to significantly more severe heart vessel inflammation and development of abdominal aorta dilations than observed in WT controls (Figure 5, B and C). LCWE injection has been shown to induce acute production of IL-1β in the peritoneal cavity at 24 hours (41), which was significantly increased in C3-deficient mice (Figure 5D). LCWE induced infiltration of F4/80^+^ macrophages into heart tissues of WT mice, which are either positive for inducible NOS (iNOS) or mannose receptor C-type 1 (MRC1) (also known as CD206), reflecting M1-like or M2-like macrophage populations, respectively (Supplemental Figure 5, A–C). A portion of these infiltrating macrophages have caspase-1 activity, marked by fluorochrome-labeled inhibitors of caspases (FLICA) (11, 43). We therefore assessed the impact of C3 deficiency on infiltrating F4/80^+^ cells that were FLICA- and NLRP3-positive in heart tissues of LCWE-injected mice. We observed that LCWE-injected *C3^–/–^* mice had significantly greater infiltration of F4/80^+^ FLICA^+^ NLRP3^+^ cells into heart tissues than WT mice (Figure 5E). These data indicate a beneficial role of C3 for the initial innate inflammatory responses in the LCWE-induced murine model of KD vasculitis.

Since the levels of C5a are acutely increased in the peritoneal lavage of LCWE-injected WT mice (Figure 5A), and given that C3 is required for C5 activation (via C5 convertase) (44), we next tested the effect of targeting the C5ar1 receptor during LCWE-induced KD. We injected WT mice with a C5ar1 antagonist, PMX205, or vehicle starting 1 day before LCWE daily until day 5 and collected tissues 2 weeks after LCWE. Similar to C3 deficiency, blocking the C5a receptor resulted in significantly more severe LCWE-induced cardiovascular lesions (Figure 5, F and G), suggesting signaling via C5ar1 is necessary to counteract the development of LCWE-induced KD vasculitis, possibly through inducing MAC formation.

To understand the contributions of the lectin and classical complement activation pathway in the development of LCWE-induced cardiovascular lesions, we next used *Mbl^–/–^* and *C1qa^–/–^* mice, which lack the MBL and classical pathways, respectively. Surprisingly, both *C1qa^–/–^* and *Mbl^–/–^* mice showed cardiovascular lesion development comparable to that of WT controls (Supplemental Figure 6, A–C). Taken together, these results suggest that although C3 and C5 activity is beneficial during LCWE-induced KD, blocking either the classical or the MBL pathways does not affect the development of cardiovascular lesions in the LCWE-induced KD model.

### C3 binds to LCWE and contributes to its clearance.

**Figure 6:**
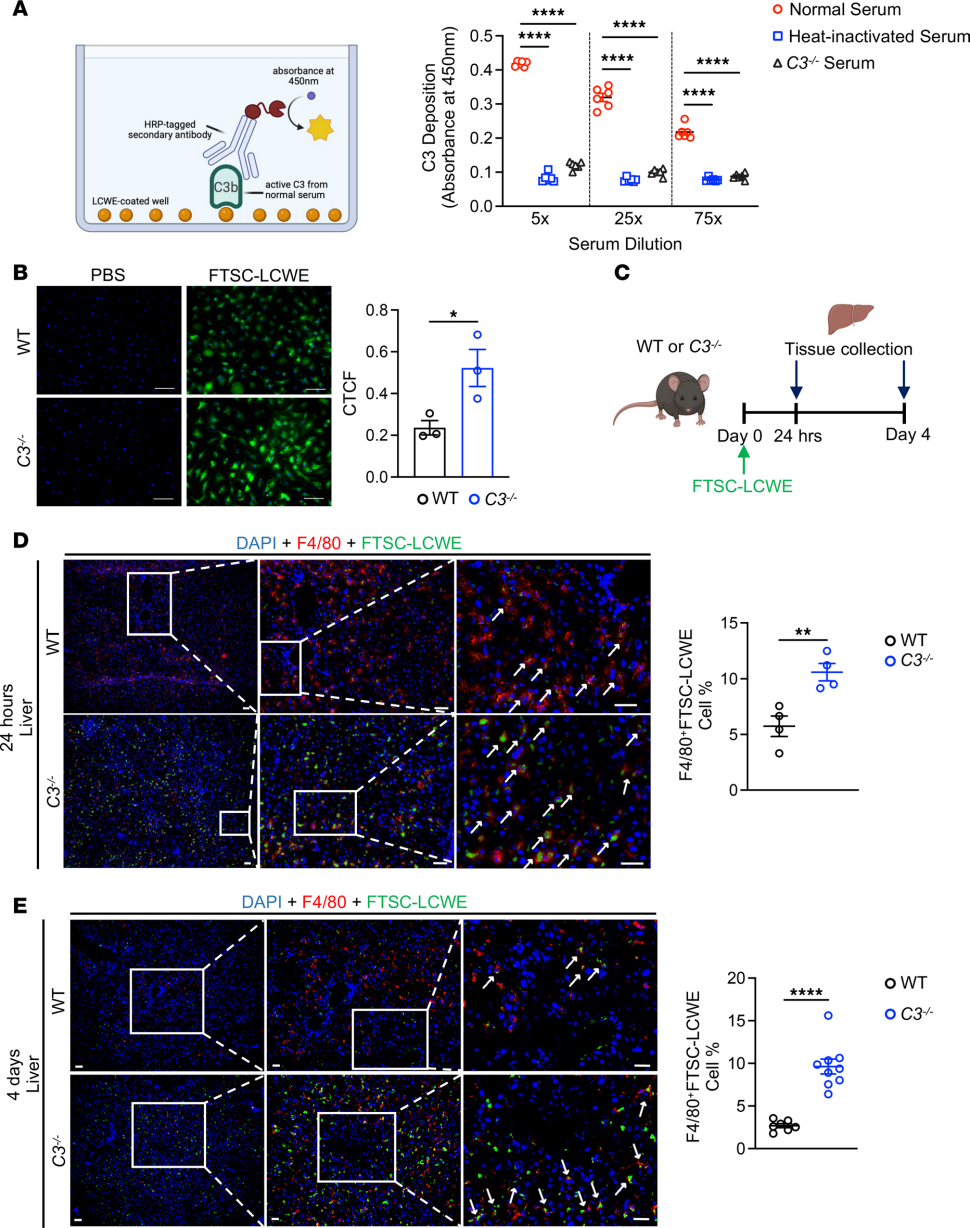
C3 binds to LCWE and is required for its clearance. (**A**) Schematic of experimental design (left). C3 binding assay to LCWE performed with normal, heat inactivated, or C3-deficient serum and absorbance measured at 450 nm (right) (*n* = 6/group). (**B**) Representative images (left) and corrected total cell fluorescence (CTCF, right) from BMDMs isolated from WT and *C3^–/–^* mice treated with PBS or FTSC-LCWE (green) for 24 hours (*n* = 3/group). DAPI (blue) was used to stain nuclei. Scale bars: 50 μm. (**C**) Schematic of experimental design for **D** and **E**. (**D**) Immunofluorescent staining (left) and quantification (right) of F4/80^+^ (red) cell numbers from livers of WT and *C3^–/–^* mice injected with FTSC-LCWE (green) at 24 hours (*n* = 4/group). DAPI (blue) was used to stain nuclei. Scale bars: 50 μm. (**E**) Immunofluorescent staining (left) and quantification (right) of F4/80^+^ (red) cell numbers from livers of WT and *C3^–/–^* mice injected with FTSC-LCWE (green) at day 4 (*n* = 7–9/group). DAPI (blue) was used to stain nuclei. Scale bars: 50 μm. Data are presented as mean ± SEM.*P < 0.05, **P < 0.01, and ****P < 0.001 by 2-way ANOVA with Tukey’s post hoc test (**A**) and unpaired 2-tailed *t* test (**B**, **D**, **E**). FTSC, fluorescein-5-thiosemicarbazide.

The C3b molecules are opsonins; they can covalently bind to pathogens (bacterial or viral) and tag them for destruction by phagocytes, such as macrophages (19). To determine whether C3 binds to LCWE, which is composed of bacterial cell wall components, LCWE-coated wells were incubated with normal, heat inactivated, or C3-deficient serum, followed by incubation with anti-C3 antibody and lastly an HRP-tagged secondary antibody (Figure 6A). Only C3 from normal serum, which has intact C3 activity, was capable of binding to coated LCWE (as evidenced by absorbance at 450 nm), whereas heat-inactivated and C3-deficient serum failed to bind to LCWE, confirming that only active C3 can deposit onto LCWE (Figure 6A). Next, we labeled LCWE with a fluorescent probe, fluorescein-5-thiosemicarbazide (FTSC) and used that to treat BM-derived macrophages (BMDMs) differentiated from WT and *C3^–/–^* mice. After 24 hours, we were able to observe FTSC-labeled LCWE engulfed by BMDMs of both genotypes (Figure 6B). Interestingly, *C3^–/–^* BMDMs showed significantly higher intracellular FTSC signal compared with WT controls in the same experiment (Figure 6B). We next injected WT and *C3^–/–^* mice with FTSC-LCWE and harvested the livers at 24 hours or 4 days after injection (Figure 6C). At both time points, *C3^–/–^* mice showed significantly higher accumulation of FTSC-LCWE in F4/80^+^ cells in their livers than WT controls (Figure 6, D and E). Overall, these results suggest that C3 binds to and contributes to the clearance of LCWE in vivo and in vitro, as C3 deficiency leads to increased deposition of LCWE into macrophages, which then leads to more severe cardiovascular lesions.

## Discussion

KD is the leading cause of acquired heart disease worldwide, and approximately 20% of patients are resistant to the standard IVIG treatment, with an increased risk of developing cardiovascular abnormalities. This highlights the urgent need for finding novel biomarkers and therapeutic tools for KD. Transcriptomic analyses and experimental studies have shown the activation of complement pathways during acute KD (21, 23, 27, 28); however, its role in the development of KD vasculitis has remained unknown. Here, we report increased expression of complement-related genes in whole blood and PBMCs of patients with acute KD. Furthermore, using a mouse model of KD vasculitis, we report increased mRNA and protein levels of complement fragments and receptors in cardiovascular lesions of LCWE-injected mice, which correlated with the peak severity of disease. Lack of C3 or blockade of C5ar1 resulted in more severe cardiovascular lesions in this murine model, whereas C1qa and Mbl were dispensable for LCWE-induced KD vasculitis. We also observed C3 binding to LCWE and confirmed that the absence of C3 impairs the clearance of labeled LCWE, leading to its accumulation in tissue-migrating macrophages.

Elevated levels and deposition of Mbl and C3 in the aortic root and coronary arteries have been observed in the candida albicans water soluble extract murine model of KD vasculitis (27). Although human studies have associated *MBL2* variants with increased risk of coronary artery lesions in patients with KD (28, 29), *Mbl* deficiency in mice did not alter the severity of LCWE-induced KD, indicating that the lectin pathway is dispensable in this context. Mbl expression was elevated in cardiovascular tissues at 2 weeks after LCWE, suggesting activation, yet alternative and classical complement pathways may potentially compensate for *Mbl* loss, reflecting a functional redundancy. Species differences in complement biology, genetic susceptibility, and the nature of the infectious trigger may further explain the observed differences between genetic associations in patients with KD and the experimental outcomes in LCWE-injected mice. Although *MBL* may modulate KD risk in patients, it is not essential for the induction of LCWE-induced KD vasculitis in mice. Furthermore, our analysis generated from publicly available datasets from whole blood of patients with acute KD and HCs indicate that genes related to the complement system are upregulated in patients with acute KD compared with convalescent KD or healthy and febrile controls. In addition, mRNA and protein levels of various complement components and receptors were increased in cardiovascular lesions associated with LCWE-induced murine KD vasculitis.

Elevated levels of C3 and its activation products are associated with chronic inflammatory states, metabolic syndrome, and cardiovascular risk factors such as atherosclerosis, insulin resistance, and endothelial dysfunction (45, 46). Of note, the generation of active C3 fragments triggers inflammatory pathways in vascular tissues, which support both the formation and destabilization of atherosclerotic plaques (47). Our finding that C3 deficiency markedly exacerbates LCWE-induced coronary arteritis suggests that an intact complement system plays a protective and regulatory role during the early immune response in this KD vasculitis mouse model. It is possible that activation of the complement pathway does not reflect a pathogenic process but rather a host-defense mechanism aimed at clearing the triggering etiologic infectious agent(s). If KD is triggered by a respiratory viral infection, as supported by epidemiological clustering, seasonality, and transcriptomic signatures (48–51), then complement-mediated opsonization, neutralization, and lytic clearance would be essential for rapid clearance of the pathogen. Supporting this concept, C3-deficient mice were not protected from the influenza virus even after being actively immunized with a cross-protective, non-neutralizing M2e (a highly conserved antigen across human influenza A subtypes) vaccine (52). Therefore, in the absence of C3, impaired viral or microbial clearance may prolong antigen persistence, sustain innate receptor stimulation, and amplify IL-1β–driven vascular inflammation, worsening coronary lesions.

Complement system fragments are increased during the acute phase of human KD (Figure 1) and can be used as markers to potentially discriminate acute KD from other febrile diseases or the convalescent phase of KD (21, 23, 27, 37–39, 53). Consistent with this, we detected higher mRNA and protein levels of the fragments and receptors in cardiovascular tissues of LCWE-injected mice at 2 weeks after injection, which correspond to the peak of LCWE-induced KD vasculitis. Strikingly, active forms of C3 and C5 (C3b and C5a) were both upregulated in the peritoneal lavage of mice starting 6 hours after LCWE, confirming an acute activation of the complement system after LCWE injection. Interestingly, we showed increased protein levels of C3 and C5 receptors as well as C3, C1qa, and Mbl in cardiovascular tissues at 2 weeks after LCWE, suggesting intracellular complement activation and the presence of tissue-infiltrating phagocytes to clear the bacterial extract in our KD model.

scRNA-Seq analysis of PBMCs from HCs and patients with acute KD showed that monocytes and neutrophils are likely the main source of complement activation. Similarly, scRNA-Seq from the abdominal aortas of LCWE-injected mice revealed infiltrating neutrophils, eosinophils, monocytes, macrophages, DCs, and B and T cells with increased levels of *C3*, *C1qa, C3ar1,* and *C5ar1* transcripts, confirming complement system upregulation in these inflammatory immune cells in LCWE-injected mice. Interestingly, fibroblasts, fibroblastic/proliferative type 2 VSMCs, and endothelial cells were significant sources of *C3* mRNA in the same dataset, suggesting that stromal cells contribute to complement activation during LCWE-induced KD vasculitis. Confirming this, spatial transcriptomics analysis showed an overlap of monocytes/macrophages and DCs with *C3*, *C1qa, C3ar1,* and *C5ar1* expression in the heart tissues of LCWE-injected mice, further supporting the pivotal role of these immune cells in complement activation during this experimental model of LCWE-induced KD vasculitis.

It is important to note that autoantibody-dependent small-vessel vasculitides, such as vasculitis associated with anti-neutrophil cytoplasmic antibody, which are pathogenically different from KD vasculitis, use complement activation to amplify vascular injury and respond to complement-targeted therapies. Indeed, therapeutic blockade of C5AR1 or anti-C5 antibody treatments were shown to have clinical benefits (54). These examples indicate that, unlike in KD where complement may facilitate clearance of the infectious trigger, complement often serves as a pathogenic effector in autoantibody-dependent vasculitis.

KD is an acute pediatric vasculitis, believed to be triggered by an infection (likely viral) in genetically susceptible hosts (2, 55). The LCWE-induced murine model recapitulates key features of KD coronary arteritis and is driven by innate immune responses to bacterial cell wall components. Our data show that genetic deficiency of C3 or inhibition of C5ar1 leads to enhanced LCWE-mediated cardiovascular lesions. In this experimental murine model of KD, complement activation may represent a helpful host defense response rather than inducer of pathology. These findings suggest a role for complement in facilitating opsonization and clearance of the inflammatory trigger and promoting resolution of innate immune activation. Importantly, this does not exclude the possibility that excessive or sustained complement activation could contribute to tissue injury, nor does it contradict clinical observations that complement activation correlates with disease activity and severity in acute KD (20, 56).

We also recognize limitations related to species and model differences. The LCWE model does not replicate all aspects of human KD, and murine complement biology may not fully mirror human disease. Accordingly, our conclusions are limited to the acute phase of experimental coronary arteritis and require validation in human disease.

IVIG is the standard therapy for KD. Among its broad immunomodulatory effects, IVIG modulates complement activity (57, 58), which does not contradict our findings, but rather emphasizes the context-dependent role of complement in vasculitis. Indeed, we hypothesize that early complement activation may contribute to protective host responses in patients with KD. However, excessive or prolonged complement activation could still contribute to vascular tissue injury. Therefore, blocking the complement system, particularly early in KD, should be approached cautiously until the stage-specific role of complement in patients with KD is better defined.

The activation of central complement proteins C3 and C5 is crucial for eliminating bacteria or viruses via opsonization and targeting pathogens for MAC (14, 59). Mice lacking C3 have been reported to exhibit increased vulnerability to gram-negative sepsis and endotoxin-induced shock (60, 61), and evidence indicates that C3a may possess antiinflammatory effects in addition to its known proinflammatory roles (61). Supporting this concept, we observed that C3 deficiency or C5ar1 blockade delayed LCWE clearance, leading to significantly increased severity of LCWE-induced cardiovascular lesions in this experimental model of KD. Surprisingly, the classical and MBL pathways were also upregulated during LCWE-induced KD. However, knockout of these complement arms individually did not affect the development of LCWE-induced cardiovascular lesions. This suggests that the lack of one of these pathways may be compensated for with the activation of the other one, or the alternative pathway, still leading to C3 and C5 activation in LCWE-induced KD. Our data also revealed that C3 deficiency results in greater acute production of IL-1β in the peritoneal fluid at 24 hours, as well as increased caspase-1 activity in the infiltrating macrophages to heart tissues at 2 weeks after LCWE, suggesting that deletion of C3 leads to diminished clearance of LCWE, which leads to increased IL-1β production and exacerbated inflammatory response in this KD vasculitis model.

Functional C3 deficiency can lead to ineffective tagging of bacterial or viral pathogens by C3b, resulting in higher bacterial loads in tissues, augmenting the inflammatory response and increasing susceptibility to infections (62–64). In line with this, C3-deficient mice display impaired generation of antigen-specific CD4^+^ and CD8^+^ T cell responses and exhibit altered cellular composition and recruitment of critical innate immune cells, including macrophages, neutrophils, and DCs, at sites of infection (65, 66). Our data show binding of C3 to LCWE. Using a fluorescently labeled LCWE (FTSC-LCWE), we were able to show that LCWE gets deposited into liver tissues at 24 hours or 4 days after LCWE injection. We detected FTSC-LCWE engulfed by F4/80^+^ cells mainly in liver tissues, which was significantly increased in *C3*^–/–^ mice, highlighting the role of C3 in phagocytosing and clearing LCWE. Notably, C3-deficiency also led to increased accumulation FLICA^+^/NLRP3^+^/F4/80^+^ cells in the heart lesions in vivo. These observations suggest that LCWE is still being engulfed by the cells, possibly via a C3-independent mechanism, such as direct recognition by TLRs. Indeed, we have previously shown the dependence of LCWE-induced KD vasculitis development on the TLR2/MyD88 axis (31).

LCWE bound selectively to C3, consistent with complement-mediated opsonization. C3b and its degradation product, iC3b, are recognized by complement receptors, particularly CR1 (CD35) and CR3 (CD11b/CD18), which are highly expressed on tissue-infiltrating monocyte-derived macrophages (67, 68). We show, in vitro and in vivo*,* that compared with WT macrophages, *C3^–/–^* macrophages accumulated LCWE intracellularly, indicating that C3 is required for efficient processing and degradation of LCWE by macrophages in tissues. Engagement of complement receptors promotes phagocytosis, phagolysosome maturation, and degradation of opsonized microbial particles (44, 67). Impaired degradation and clearance of LCWE by *C3^–/–^* macrophages may lead to accumulation and persistence of intracellular LCWE, which may continuously activate the NLRP3 inflammasome and promote sustained innate immune activation. In LCWE-induced KD vasculitis, coronary lesions are enriched in inflammatory iNOS^+^ (M1-like) macrophages, and many of the infiltrating macrophages are NLRP3^+^ and caspase-1 active, indicating inflammasome activation. We therefore propose that complement activation promotes an early and efficient macrophage-mediated clearance of LCWE, limiting the duration of innate immune cell stimulation by LCWE. In the absence of C3 or with C5ar1 blockade, however, impaired clearance of LCWE and its accumulation in macrophages may contribute to persistent innate immune activation and exacerbate vascular inflammation.

Our data demonstrate that complement facilitates LCWE tissue clearance, as C3 deficiency worsened the severity of LCWE-induced KD and led to increased accumulation of FTSC-labeled LCWE in tissue macrophages. Prior studies have shown that macrophages are essential for the development of LCWE-induced cardiovascular lesions, as clodronate-mediated depletion prevents coronary arteritis (11), and that NLRP3 inflammasome activation and IL-1β production are critical downstream mediators of vascular inflammation in this experimental KD vasculitis model (10, 11, 41). Here, we observed that C3 deficiency in mice led to the accumulation of macrophages in heart tissues and accumulation of LCWE in macrophages. These observations and our previous findings indicate that complement-mediated clearance of LCWE may provide a mechanistic link by limiting inflammasome activation in tissue-recruited inflammatory macrophages, although direct causal evidence that macrophage engulfment of LCWE drives KD vasculitis remains to be determined.

Based on our data, we propose that the complement system facilitates opsonization and clearance of LCWE-derived microbial components or potential viral triggers, reducing antigen persistence in vascular tissues and limiting coronary and aortic inflammation in LCWE-induced cardiovascular lesions. In the absence of C3, impaired clearance of bacterial components in LCWE allows continued stimulation of pathogen pattern receptors and innate immune cells, including macrophages, leading to NLRP3 inflammasome activation and IL-1β production. This sustained IL-1β–driven response further promotes the recruitment of monocytes/macrophages and neutrophils, amplifying vascular injury and coronary artery lesions in this experimental vasculitis model. Thus, the complement system does not act solely as a proinflammatory amplifier but also regulates NLRP3/IL-1β–mediated vascular inflammation and tissue damage in this murine model of KD vasculitis via the clearance of triggering microbial components. Our data provide a mechanistic link between the complement pathway and its roles in pathogen clearance, NLRP3 activation, and IL-1β during LCWE-induced KD vasculitis and suggest that dysregulation at any step may exacerbate inflammation in cardiovascular tissues. Our data support a broader model in which regulated complement activity restrains, rather than promotes, coronary arteritis by eliminating the upstream microbial trigger in KD. Future studies should examine whether specific complement signatures (C3a, iC3b/C3d, C5a, Ba/Bb, sC5b-9) in patients with KD correlate with IVIG response and coronary outcomes.

## Methods

### Sex as a biological variable.

Our study exclusively examined male mice. It is unknown whether the findings are relevant for female mice. It is previously published that LCWE injection induced more robust and more consistent coronary vasculitis and abdominal aorta aneurysms in males compared with female mice (9, 11). This effect is similar to observations in human KD, where there is a 1.5:1 male-to-female ratio. Because of the low disease incidence in female mice, larger groups of female mice would be required to show the difference with adequate statistical power between PBS- and LCWE-injected groups. In contrast, only groups of 10 male mice are required.

### Mice.

All procedures were approved by the IACUC of Cedars-Sinai Medical Center. Mice were housed under specific pathogen–free conditions at Cedars-Sinai Medical Center. Mice were kept at 22°C under a 12-hour light/12-hour dark cycle and fed a standard chow diet and water ad libitum. WT C57BL/6J*,* *C3^–/–^* (strain 029661), *C1qa^–/–-^* (strain 031675), and *Mbl^–/–^* (strain 006122) mice were obtained from The Jackson Laboratory. Five-week-old male mice were used for experimental purposes because LCWE injection induces stronger and more consistent coronary vasculitis lesions and abdominal aorta dilations in male mice than female mice (9, 69). Mice were randomly assigned to the different experimental groups.

### The LCWE-induced KD vasculitis murine model.

*Lactobacillus casei* (ATCC; 11578) cell wall extract was prepared as previously described (10). Five-week-old male mice were injected i.p. with 500 μL of LCWE or PBS (control group). One or 2-weeks after injection, mice were euthanized, and blood was collected. Hearts and aortas were harvested and embedded in OCT (Sakura Finetek) compound for histology. Abdominal aortas were photographed after dissection and then embedded in OCT. Maximal abdominal aorta diameter was calculated by measuring 5 different areas on the infrarenal section with ImageJ (NIH). Representative images were selected based on corresponding inflammatory scores or aortic measurements, reflecting the mean of the sample group.

### FTSC labeling of LCWE.

First, a final concentration of 10 mM sodium periodate (Acros Organics; 419610050) was added to 10 mL of LCWE and incubated for 30 minutes in the dark at room temperature. Second, a final concentration of 10 mM of sodium bisulfite (Acros Organics; 419440050) was added to LCWE and incubated in the dark for 15 minutes at room temperature. Next, 2 mg/mL FTSC (MilliporeSigma; 46985) was added and incubated overnight at 4°C on a shaker. The next day, 25 mM sodium cyanoborohydride (Alfa Aesar; 87839) was added and incubated at 37°C on a shaker for 2.5 hours. Last, the LCWE was dialyzed against 3 liters of PBS overnight at 4°C, aliquoted, and stored at –80°C.

### Tissue fixation, H&E staining, and histological analysis.

First, 7 μm-thick serial cryosections from OCT-embedded heart tissues were stained with H&E (Sigma-Aldrich) for histological analysis or were used for immunofluorescent staining. Histopathological examination and inflammatory scoring of heart tissues were performed from these sections by a senior investigator blinded to the experimental groups. Heart vessel inflammatory score (coronary arteritis, aortic root vasculitis, and myocarditis) was determined as previously described (10). Only heart sections showing the coronary artery branch separating from the aorta were scored. Briefly, acute inflammation, chronic inflammation, and connective tissue proliferation were each assessed using the following scoring system: 0 = no inflammation, 1 = rare inflammatory cells, 2 = scattered inflammatory cells, 3 = diffuse infiltrate of inflammatory cells, and 4 = dense clusters of inflammatory cells. Fibrosis was determined using the following scoring system: 0 = no medial fibrosis, 1 = medial fibrosis involving less than 10% of the coronary artery branch circumference, 2 = medial fibrosis involving 11% to 50% of the coronary artery branch circumference, 3 = medial fibrosis involving 51% to 75% of the coronary artery branch circumference, and 4 = medial fibrosis involving more than 75% of the coronary artery branch circumference. All 4 scores were combined to generate a severity score called the heart vessel inflammation score, as previously published (10). Pictures were taken using a Biorevo BZ-9000 or BZ-X710 microscope (Keyence). Representative images were chosen based on corresponding inflammatory scores that were closest to the mean of the sample group.

### IL-1β, C3b, and C5a quantification from mouse peritoneal lavage.

Mice were injected with either PBS or LCWE as described above. Peritoneal lavage was collected in sterile PBS 6 or 24 hours later, centrifuged at 400*g* at 4°C for 5 minutes, and the cells were discarded. C3b (MyBioSource; MBS269569), C5a (MyBioSource; MBS2021453), or IL-1β (R&D Systems; mouse IL-1β/IL-1F2 DuoSet ELISA; DY401) concentration was assessed from the supernatants by ELISA according to the manufacturer’s protocol.

### In vivo PMX205 treatment.

WT mice were injected daily with either DMSO or PMX205 (2 mg/kg) i.p. starting 1 day before LCWE injection until day 5. Mice were euthanized at 2 weeks after LCWE, and tissues were harvested and analyzed as described above.

### BMDM isolation and culture.

BM was collected from bilateral tibia and femurs of mice with RPMI containing 1% penicillin-streptomycin cocktail and filtered through a cell strainer (BD Biosciences, 352350). Cells were centrifuged at 300*g* for 5 minutes and resuspended in RPMI enriched with 20% L929 cells conditioned medium, 10% FBS, and 1% penicillin-streptomycin cocktail, followed by growth on petri dishes and differentiation to macrophages for 7 days. After differentiation, cells were treated with LCWE (60 μg/mL) or FTSC-LCWE (60 μg/mL) for 24 hours.

### RNA isolation and qRT-PCR.

Total RNA was isolated by TRIzol Reagent (Invitrogen; 15596018) according to the manufacturer’s protocol, and reverse-transcribed by using RevertAid First Strand cDNA synthesis kit (Thermo Fisher Scientific; K1691) to cDNA, according to the manufacturer’s protocol. cDNA was amplified using specific primers specified below and Power-Up-SYBR green (Applied Biosystems; A25742) on a CFX96 Real-Time System (BioRad). Gene expression was quantified using triplicate samples with the relative threshold ΔΔCt method: ΔΔCt = (primer efficiency) ^(−ΔΔCt)^, where ΔΔCt means ΔCt (target gene) − ΔCt (reference gene).

### Protein lysates, SDS/PAGE electrophoresis, transfer, and Western blotting.

Abdominal aorta tissues were homogenized in RIPA buffer (0.5 M Tris-HCl, pH 7.4, 1.5 M NaCl, 2.5% deoxycholic acid, 10% NP-40, 10 mM EDTA; MilliporeSigma; 20-188). Lysates were cleared with brief centrifugation for 10 minutes at 8,000*g*, normalized, and boiled at 95°C after the addition of 6× SDS loading dye. Proteins were then loaded to gradient SDS/PAGE gels and transferred to nitrocellulose or PVDF membranes. Membranes were subject to blocking (1 hour at room temperature) and primary antibody (overnight at 4°C; 1:2,000 dilution) against C3 (Proteintech; 21337-1-AP), Mbl (R&D Systems; AF2077-SF), or β-actin (Santa Cruz Biotechnology; sc-47778) followed by secondary antibody (Peroxidase AffiniPure goat anti-rabbit IgG H+L; Jackson ImmunoResearch; 1:10,000) incubation at room temperature for 1 hour in 5% (w/v) dry milk or BSA in tris-buffered saline buffer with 0.1% Tween-20 (v/v) on a shaker. Membranes were developed in ECL prime reagent (Thermo Fisher Scientific; 34577) and images were captured with ChemiDoc Imager (BioRad). Blots shown are representative of 2 experiments. Quantifications were performed with Image Lab software (BioRad).

### Immunofluorescent staining.

First, 7 μm-thick sections were obtained from OCT frozen heart, abdominal aorta, or livers, fixed in ice-cold acetone for 5 minutes, washed with PBS, and blocked for 1 hour at room temperature with 3% donkey serum (Abcam; ab7475) in PBS. Sections were then incubated with primary C3 (Proteintech; 21337-1-AP), Mbl (R&D Systems; AF2077-SF), C1qa (Proteintech; 11602-1-AP), C3ar1 (MyBioSource; MBS584674), C5ar1 (Abcam; ab117579), F4/80 (Tonbo Biosciences; 70-4801 or BioLegend; 23122), NLRP3 (Invitrogen; 768319), anti-mouse iNOS antibody conjugated with FITC (clone 11F6, BD Biosciences; 610330), and anti-mouse MRC1 rabbit antibody (clone E6T5J, Cell Signaling Technology; 24595) antibodies or isotype controls (1:100 dilution) in blocking solution overnight at 4°C in a humid chamber. The next day, sections were washed again with PBS, incubated for 1 hour at room temperature with fluorescently labeled appropriate secondary antibodies prepared in blocking solution, and then washed again with PBS. Caspase-1 activity in tissues was detected using FLICA (Immunochemistry Technologies; SKU 98), according to the manufacturer’s protocol. Slides were then mounted with Fluoroshield mounting medium containing DAPI (Abcam; ab104139). Images were obtained using a Biorevo BZ-X710 (Keyence) fluorescent microscope and were further analyzed with ImageJ (NIH) software. Corrected total cell fluorescence (CTCF) was calculated with the following formula: CTCF = integrated density – (area of selected cell × mean fluorescence of background readings). Cells expressing both F4/80^+^ and iNOS^+^ (M1-like macrophages), as well as those expressing both F4/80^+^ and MRC1^+^ (M2-like macrophages), were identified and quantified using QuPath’s cell detection function. Parameters were set to detect cells based on size, shape, and fluorescence intensity thresholds specific to the fluorescent dyes used. The accuracy of cell detection was verified by manually inspecting randomly selected fields of view. Data are presented as the total number of single-positive and double-positive cells per total lesion area of each section. For all immunofluorescent staining, isotype controls for all primary antibodies were used to distinguish positive staining from the background.

### Analysis of human and mouse gene expression datasets.

Publicly available gene expression datasets GSE68004 (37), GSE73461 (38), GSE178491 (39), and GSE141072 (9) were obtained from NCBI’s GEO. Transcriptomic datasets were accessed and loaded into R via GEOQuery, and summary statistics were generated with limma topTable function. KEGG’s Complement and Coagulation Cascades gene set (hsa04610) was used as a reference to analyze the expression of complement-related genes. DEGs (Benjamini-Hochberg adjusted *P* < 0.05 and fold-change > 1.5) from the gene signatures were identified in these datasets with the R package pheatmap.

### Analysis of human scRNA-Seq dataset.

The PBMC scRNA-Seq dataset GSE168732 (40) was accessed from NCBI’s GEO, which contains samples from patients with KD before IVIG treatment (*n* = 6) and healthy control patients (*n* = 3). Preprocessing, integration, and analysis were carried out using the R package Seurat (v5.1.0). Quality control was performed to remove low-quality cells and genes. The remaining cells’ data was normalized and scaled via LogNormalize() and ScaleData(), respectively, identifying the top 2,000 variable features with FindVariableFeatures(). Dimensional reduction was performed on the normalized data by principal component analysis (PCA) with RunPCA() function (npcs = 50), and nearest neighbor graph construction was created via FindNeighbors(). Doublet removal by sample via R package DoubletFinder was carried out before integration. Samples were integrated with Harmony via Seurat’s function RunHarmony() to remove batch effect. FindNeighbors() for nearest neighbor graph construction was performed again after integration, as well as RunUMAP() to generate a visual projection of the integrated data. Cells were then clustered utilizing Seurat’s FindClusters() function with the Leiden algorithm (algorithm = 4, resolution = 4, method = igraph). Differential of markers in clusters was calculated using FindAllMarkers() (method = wilcox, min.pct = 0.25, only.pos = T), annotating clusters based on their differential expression of canonical cell-type markers (log_2_ fold change > 1 and adjusted *P* [FDR] < 0.05).

### Analysis of scRNA-Seq dataset of mouse abdominal aortas and spatial transcriptomics of hearts.

Expression of *C3, C1qa, C3ar1,* and *C5ar1* was assessed in a previously generated and publicly available scRNA-Seq dataset from the abdominal aorta tissues of PBS-injected (*n* = 9 tissues pooled) and LCWE-injected (*n* = 6 tissues pooled) mice (GSE178765) (41). Uniform manifold approximation and projection (UMAP) was performed using the R package UMAP v0.2.2.0 (70); cell clustering and annotations were performed with Seurat V3 and SingleR (41). The spatial expression of *C1qa*, *C3ar1*, *C5ar1,* and *C3* were analyzed in a previously generated Visium (10x Genomics, Inc.) dataset from heart tissues of PBS- and LCWE-injected mice (GSE178799) via the established pipeline in the published dataset (41).

### C3 deposition assay.

Wells were incubated with LCWE at 4°C overnight. The next day, wells were washed (0.05% Tween-20 in PBS), blocked (1% BSA in PBS) for 1 hour at room temperature, followed by incubation with normal, heat-inactivated, or C3-deficient serum for 1 hour. Wells were then incubated with anti-C3 antibody (Abcam, ab97462; 0.5 μg/mL in 1% BSA in PBS) followed by incubation with HRP-tagged secondary antibody (1:750 dilution). Wells were washed again, and absorbance was measured at 450 nm to determine C3 binding to LCWE.

### Statistics.

GraphPad Prism 10 or R software was used to statistically analyze data and create graphs. Statistical analyses used for each figure panel are indicated in the figure legends. For comparisons of 2 groups, a 2-tailed unpaired Student’s *t* test, with Welch’s correction when indicated, was used for normally distributed data. For nonparametric data, the Mann-Whitney 2-tailed *U*/Wilcoxon’s rank-sum test was used. For comparison of more than 2 groups, 1-way ANOVA with Tukey’s posttest analysis was used for normally distributed data. The Kruskal-Wallis test with Dunn’s multiple-comparison test was used for nonnormally distributed data. A 2-way ANOVA was used when there were 2 independent variables with multiple groups, such as genotype and treatment. Results are reported as mean ± SEM, where each point represents 1 sample. A *P* value less than 0.05 was considered statistically significant. No statistical methods were used to predetermine the sample size.

### Study approval.

Studies were approved by the IACUC at Cedars-Sinai Medical Center, Los Angeles, California, USA, and were conducted in accordance with institutional guidelines and NIH policies for the care and use of laboratory animals.

### Data availability.

All data related to the findings are present in the paper or in the Supporting Data Values file.

## Author contributions

AEA, BK, and MA conceived the project and designed the experiments. AEA, BK, EAA, RAP, TTC, TN, and YL performed the experiments. AEA, BK, BLR, and RAP analyzed the data. MNR helped conceptualize and discuss results. AEA, MNR, and MA wrote the article with input from all authors.

## Conflict of interest

The authors have declared that no conflict of interest exists.

## Funding support

This work is the result of NIH funding, in whole or in part, and is subject to the NIH Public Access Policy. Through acceptance of this federal funding, the NIH has been given a right to make the work publicly available in PubMed Central.

- American Heart Association 24POST1196203 (to AEA).
- NIH R01-HL139766 (to MNR).
- NIH R01-HL170580 (to MA).

## Supplementary Material

Supplemental data

Unedited blot and gel images

Supporting data values

## Version 1 05/12/2026

In-Press Preview

## Version 2 07/22/2026

Electronic publication
