# Supplementary material for: Protective role of complement signaling in Kawasaki disease vasculitis
Source: JCI Insight. 2026 May 12;11(14):e203997. doi: 10.1172/jci.insight.203997 (PMC13461170; doi:10.1172/jci.insight.203997)
Supplement: Supplementary file 1 [file jciinsight-11-203997-s127.pdf]

Figure S1

A

Human	
A2M	*F10*
BDKRB1	*F11*
BDKRB2	*F12*
C1QA	*F13A1*
C1QB	*F13B*
C1QC	*F2*
C1R	*F2R*
C1S	*F3*
C2	*F5*
C3	*F7*
C3AR1	*F8*
C4A	*F9*
C4B	*FGA*
C4BPA	*FGB*
C4BPB	*FGG*
C5	*KLKB1*
C5AR1	*KNG1*
C6	*MASP1*
C7	*MASP2*
C8A	*MBL2*
C8B	*PLAT*
C8G	*PLAU*
C9	*PLAUR*
CD46	*PLG*
CD55	*PROC*
CD59	*PROS1*
CFB	*SERPINA1*
CFD	*SERPINA5*
CFH	*SERPINC1*
CFI	*SERPIND1*
CPB2	*SERPINE1*
CR1	*SERPINF2*
CR2	*SERPING1*
	TFPI
	THBD
	VWF

B

Mouse	
A2m	*F13b*
Bdkrb1	*F2*
Bdkrb2	*F2r*
C1qa	*F3*
C1qb	*F5*
C1qc	*F7*
C2	*F8*
C3	*F9*
C3ar1	*Fga*
Hc	*Fgb*
C5ar1	*Fgg*
C6	*Klkb1*
C7	*NA*
C8a	*Masp1*
C8b	*Masp2*
C8g	*Mbl2*
C9	*Plat*
Cd46	*Plau*
NA	*Plaur*
Cd59b	*Plg*
Cfb	*Proc*
Cfd	*Pros1*
Cfh	*NA*
Cfi	*Serpina5*
Cpb2	*Serpinc1*
NA	*Serpind1*
Cr2	*Serpine1*
F10	*Serpinf2*
F11	*Serping1*
F12	*Tfpi*
F13a1	*Thbd*
	Vwf

Figure S1. ***List of reference genes for the human and mouse analyses.*** **(A-B)** KEGG's *"Complement and Coagulation Cascades"* gene set (hsa04610) was used as reference to analyze the expression of complement related genes from GSE68004, GSE73461, GSE178491 datasets from human (A) whole blood and GSE141072 dataset from mouse (B) abdominal aortas. A total of 69 genes were identified with 59 murine orthologs.

Figure S2

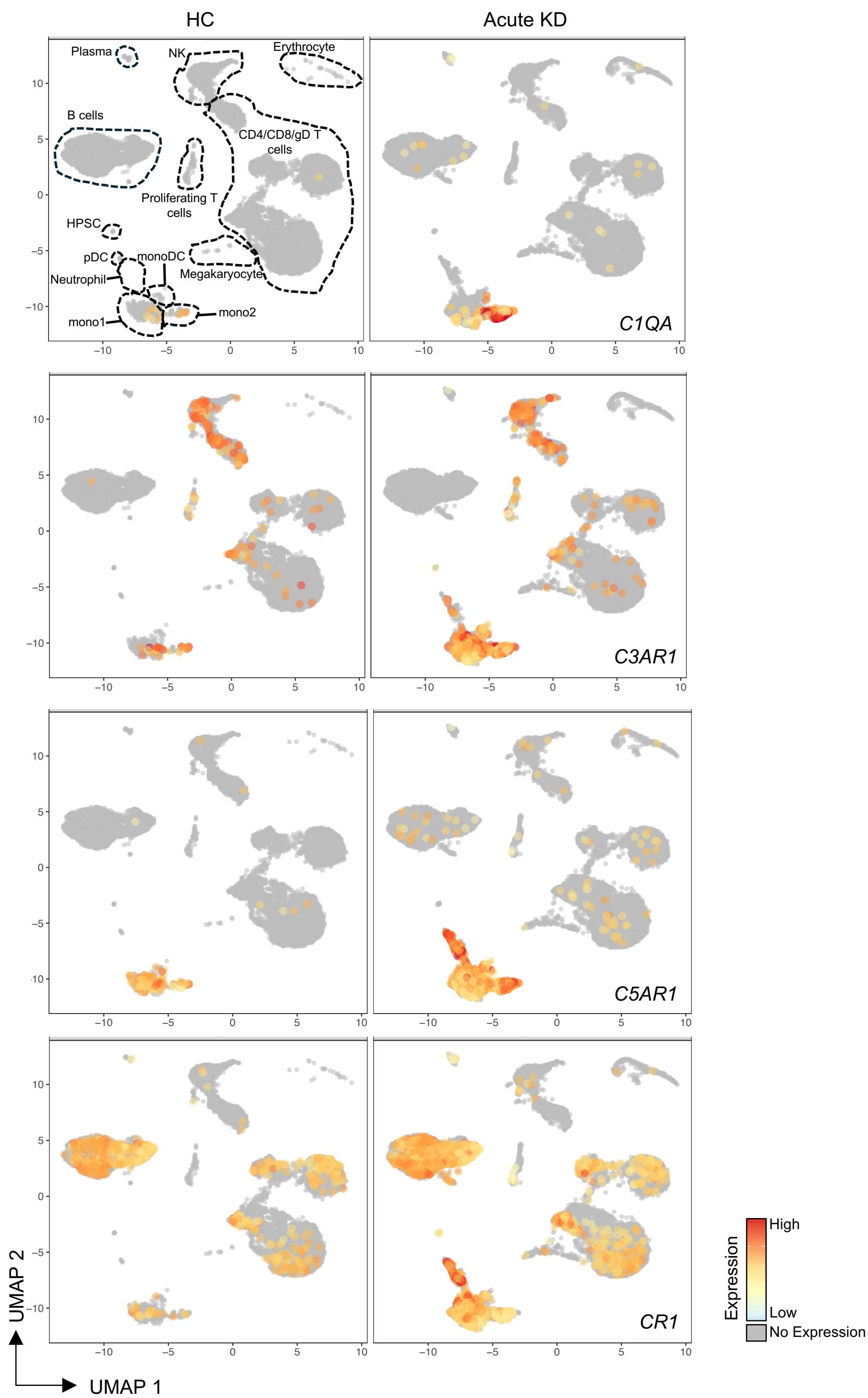

Figure S2. ***Increased cellular expression of selected genes from the complement pathway in acute KD.*** UMAP visualization split to show separated visualization of the scRNA-seq datasets from the peripheral blood mononuclear cells of healthy controls (HC) or acute KD patients (GSE168732) representing a gradient expression of selected genes related to complement pathway activation. NK: natural killer cells, HPSC: hematopoietic stem and progenitor cells, pDC: plasmacytoid dendritic cells, mono: monocyte.

Figure S3

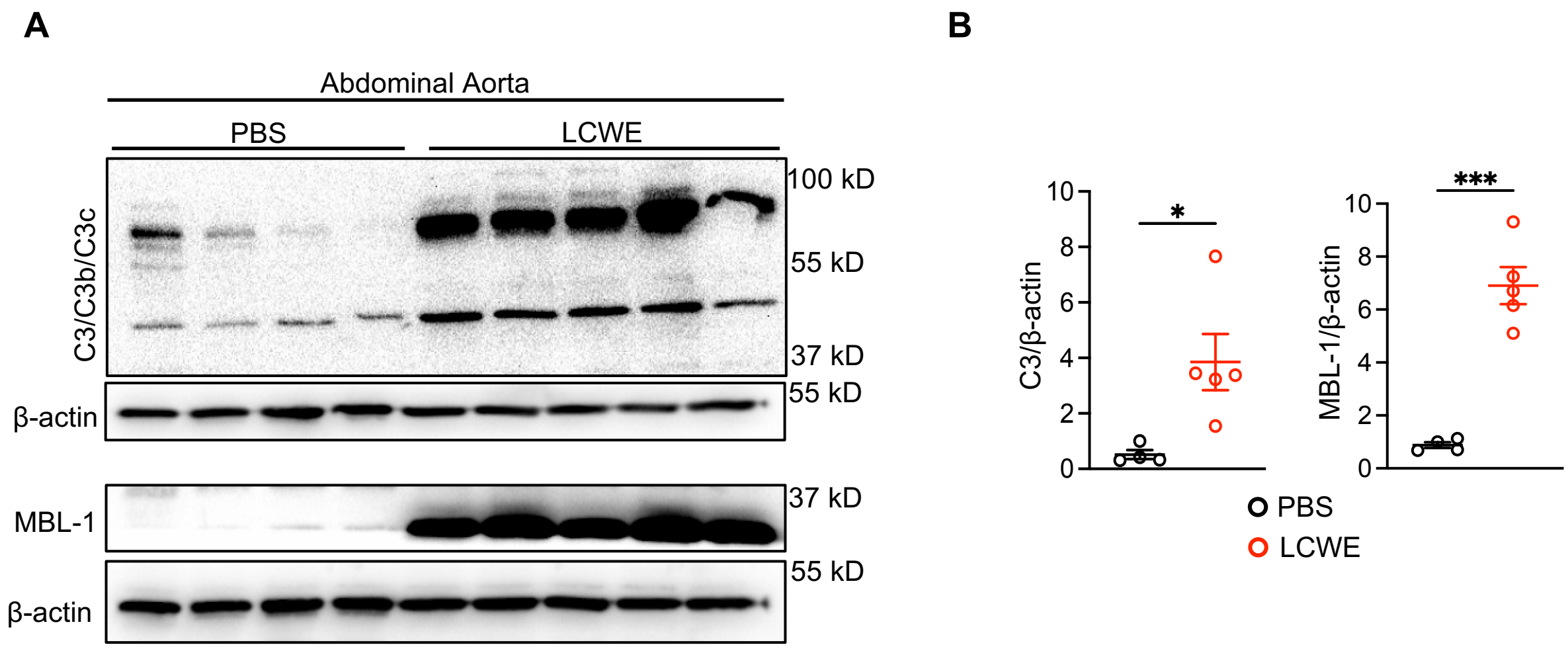

Figure S3. ***Complement pathway expression in cardiovascular tissues of LCWE-induced KD mice.*** **(A-B)** Western blot (WB) analysis (A) and quantification of band intensities (B) of C3/C3b/C3c and Mbl-1 levels from abdominal aortas of PBS or LCWE-injected mice at 2 weeks post-injection, normalized to β-actin (n=4,5/group). Data are presented as mean ± SEM. **p-value* < 0.05, ***p-value* < 0.01, ****p-value* < 0.001, and *****p-value* < 0.001 by unpaired t-test.

Figure S4

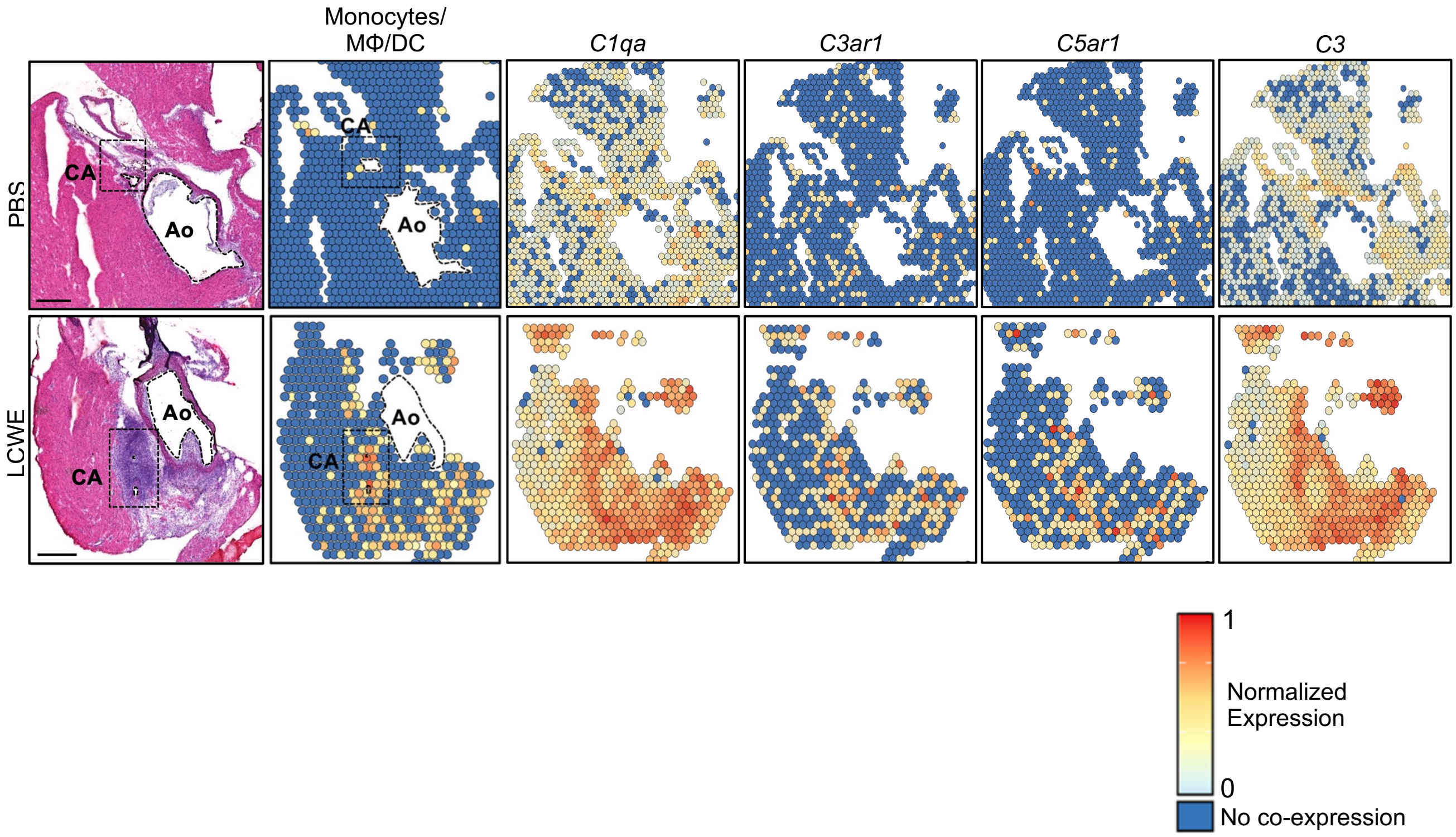

Figure S4. ***Spatial transcriptomics analysis of complement pathway related genes in heart tissues of PBS- or LCWE-injected mice.*** Representative H&E-stained heart sections from PBS (top) and LCWE-injected (bottom) mice and overlaid expression of genes defining monocytes/macrophages/DC (*Csfr1, Cd14, Itgax, Itgam, Fcgr3*) and expression of C1qa, C3ar1, C5ar1 and C3 at 2 weeks post-injection (GSE178799). Scale bars, 500µm. Dark blue: no co-expression. Light blue-yellow-red gradient: min-max normalization of counts per 10,000 reads expression. H&Es and UMAP of monocytes/macrophages/DC are reproduced from ref. 42. CA: coronary artery, Ao: Aorta, DC: dendritic cell, Mφ: macrophage.

Figure S5

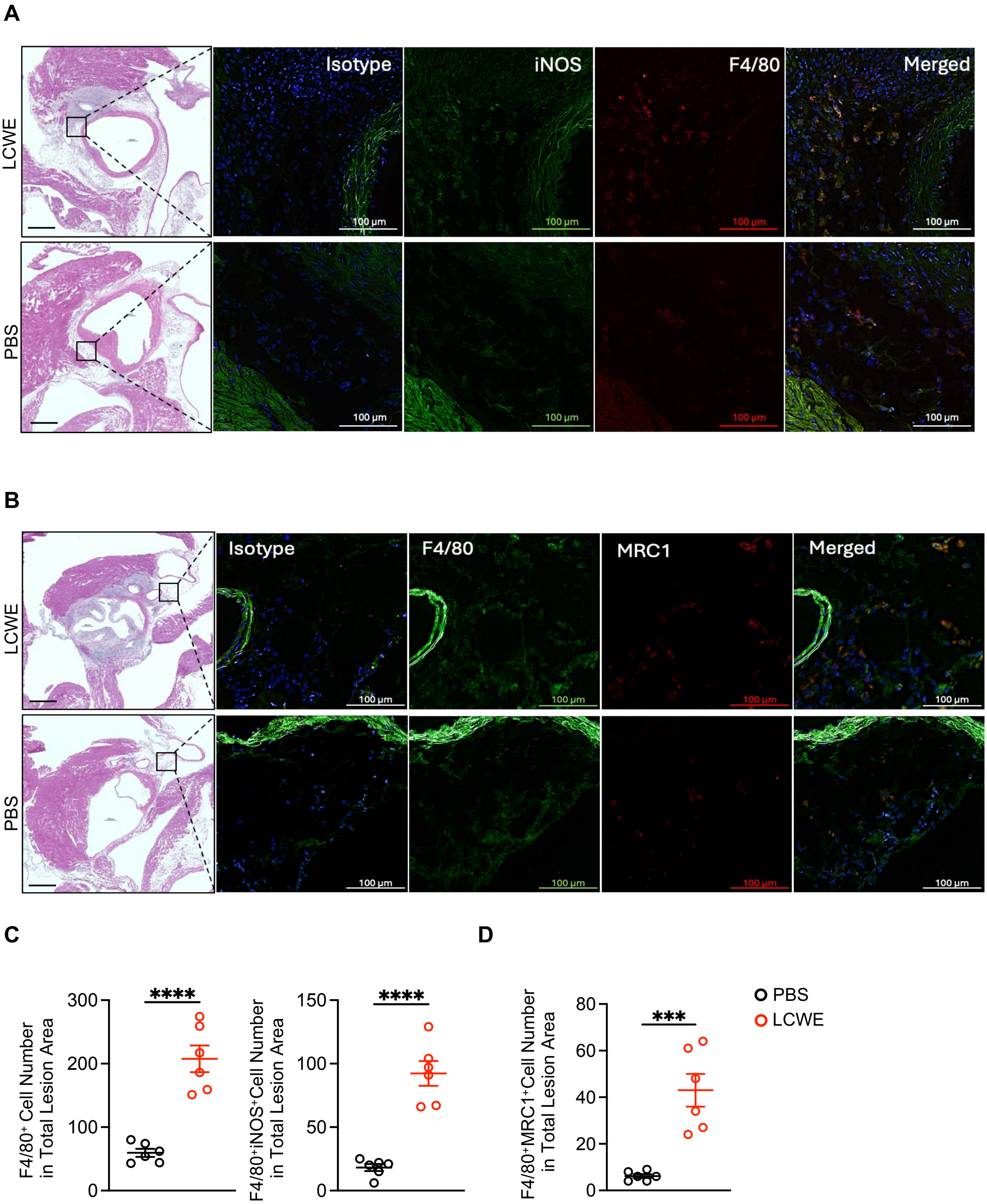

Figure S5. ***LCWE induces the infiltration of M1 and M2-like macrophages into heart tissues.***
(A) Representative H&E staining and immunofluorescence images F4/80 (red) and iNOS (green) from WT mice injected with PBS or LCWE at 2 weeks post-injection. **(B)** Representative H&E staining and immunofluorescence images F4/80 (green) and MRC1 (red) from WT mice injected with PBS or LCWE at 2 weeks post-injection. **(C)** Quantification of the number of F4/80-positive F4/80/iNOS-double positive cells in the total lesion area in WT mice injected with PBS or LCWE from panel A. **(D)** Quantification of the number and MRC1 double-positive cells in the total lesion area in WT mice injected with PBS or LCWE from panel B. Scale bars: 500 µm (H&E) and 100 µm (immunofluorescent stainings). DAPI is used to stain nuclei. Each symbol represents 1 section for a total of 6 sections obtained from 3 mice per group. Data are presented as mean ± SEM. **p-value* < 0.05, ***p-value* < 0.01, ****p-value* < 0.001, and *****p-value* < 0.001 by unpaired t-test (C, D).

Figure S6

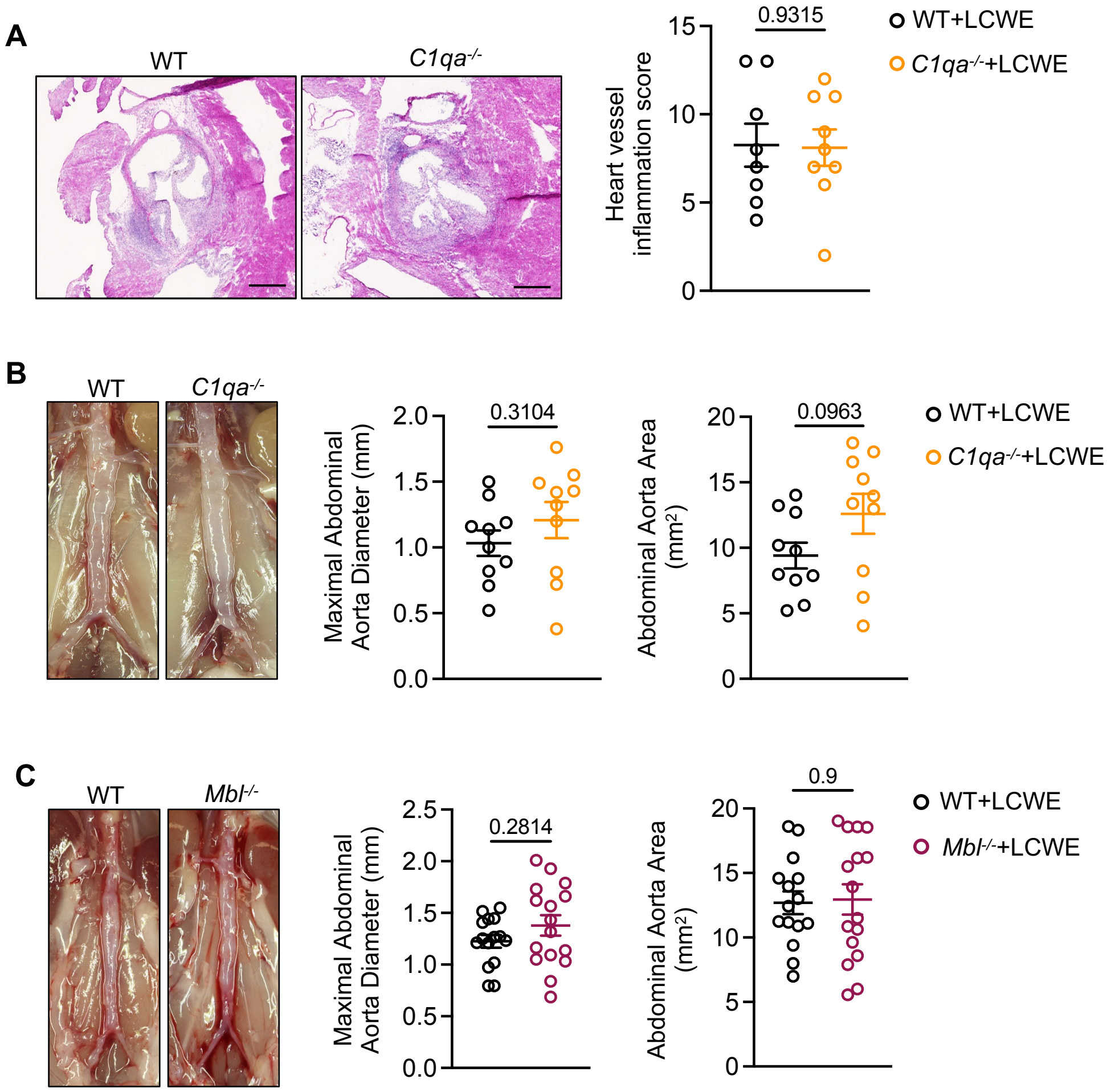

Figure S6. *Classical or the mannose-binding lectin pathways are dispensable for the development of cardiovascular lesions in LCWE-induced murine KD vasculitis.* (A) Representative H&E-stained heart sections and heart vessel inflammation scores of LCWE-injected WT and *C1qa*$^{-/-}$ mice, at 2 weeks post-injection (n=8,9/group). Scale bars, 500μm. **(B)** Representative pictures of the abdominal aorta areas, maximal abdominal aorta diameter and abdominal aorta area measurements of LCWE-injected WT and *C1qa*$^{-/-}$ mice at 2 weeks post-injection(n=10/group). **(C)** Representative pictures of the abdominal aorta areas, maximal abdominal aorta diameter and abdominal aorta area measurements of LCWE-injected WT and *Mbl*$^{-/-}$ mice at 2 weeks post-injection (n=15,16/group). Data are pooled from 2 separate experiments (A-C) and presented as mean ± SEM. **p-value* < 0.05, ***p-value* < 0.01, ****p-value* < 0.001, and *****p-value* < 0.001 by unpaired t-test (A, B, C).
