# Supplementary material for: Protective role of complement signaling in Kawasaki disease vasculitis
Source: JCI Insight. 2026 May 12;11(14):e203997. doi: 10.1172/jci.insight.203997 (PMC13461170; doi:10.1172/jci.insight.203997)
Supplement: Supplementary file 2 [file jciinsight-11-203997-s128.pdf]

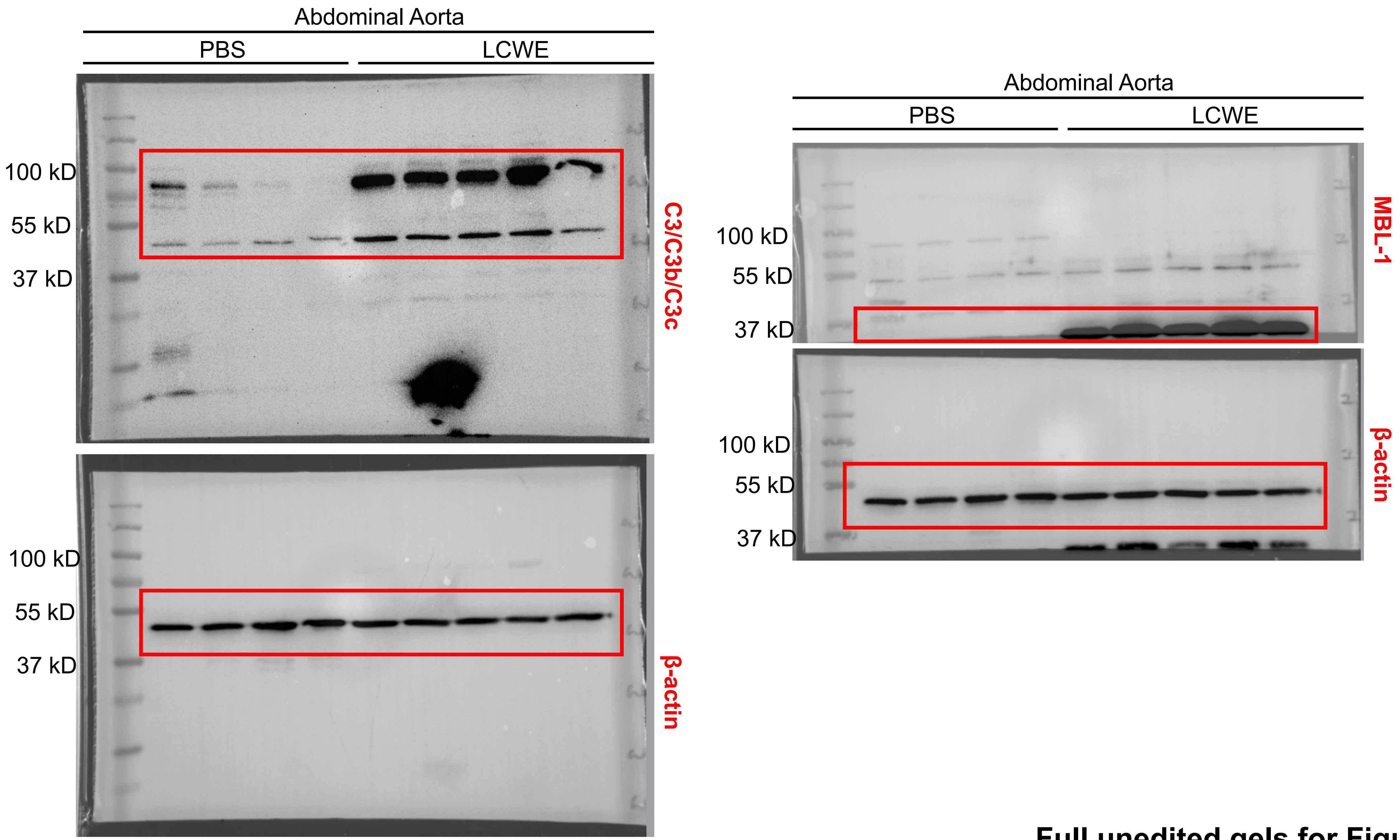

Full unedited gels for Figure S3A
